# Emotional coping strategies in children with and without special educational needs during the COVID-19 pandemic in Saudi Arabia

**DOI:** 10.3389/fpubh.2025.1564307

**Published:** 2025-09-02

**Authors:** Shuliweeh Alenezi, Mohammed Alarabi, Ahmed S. Alyahya, Ahmad H. Almadani, Afnan Almarshedi, Maha Algazlan, Faisal Alnemary, Fahad A. Bashiri, Samah Hazem Alkhawashki, Maram Hani Altuwariqi, Rafif Alsedrani, Aqeel Alkhiri, Sultan A. Abughanim, Addo Boafo, Mohamad-Hani Temsah

**Affiliations:** ^1^Department of Psychiatry, College of Medicine, King Saud University, Riyadh, Saudi Arabia; ^2^Department of Psychiatry, King Saud University Medical City, King Saud University, Riyadh, Saudi Arabia; ^3^SABIC Psychological Health Research and Applications Chair (SPHRAC), Department of Psychiatry, College of Medicine, King Saud University, Riyadh, Saudi Arabia; ^4^Department of Neurosciences, King Abdullah bin Abdulaziz University Hospital, Princess Nourah bint Abdulrahman University, Riyadh, Saudi Arabia; ^5^Psychiatry Department, Prince Sultan Military Medical City, Riyadh, Saudi Arabia; ^6^Clinical Sciences Department, College of Medicine, Princess Nourah bint Abdulrahman University, Riyadh, Saudi Arabia; ^7^Autism Center of Excellence, Riyadh, Saudi Arabia; ^8^Pediatric Neurology Division, Department of Pediatrics, College of Medicine, King Saud University, Riyadh, Saudi Arabia; ^9^Department of Psychiatry, King Fahad Armed Forces Hospital, Jeddah, Saudi Arabia; ^10^Mental Health Program, Children's Hospital of Eastern Ontario, Ottawa, ON, Canada; ^11^Department of Psychiatry, University of Ottawa, Ottawa, ON, Canada; ^12^Pediatric Department, King Saud University Medical City, King Saud University, Riyadh, Saudi Arabia

**Keywords:** coping strategies, children with special educational needs, typically developing children, COVID-19 pandemic, Saudi Arabia, maladaptive coping, adaptive coping, mental health outcomes

## Abstract

**Introduction:**

The COVID-19 pandemic significantly disrupted children's daily lives, especially those of children with special educational needs and disabilities (SEND). This study aimed to compare the coping strategies of children with SEND to those of typically developing (TD) peers, as reported by their parents, and to identify the factors associated with coping efficacy early during the COVID-19 pandemic.

**Methodology:**

We conducted a nationwide cross-sectional survey between May and July 2020 using the Arabic translation of a global project's survey. Participants were recruited from all regions of Saudi Arabia through text messages sent to beneficiaries of the Ministry of Human Resources and Social Development, the Autism Center of Excellence, and the Authority for Persons with Disabilities. Parents of 548 pairs of SEND and TD children, matched by age (±3 years), completed the survey and were included in the analysis. Coping strategies were analyzed and grouped into adaptive and maladaptive factors.

**Results:**

TD children and children with SEND were aged, on average, 9.12 ± 3.95 years and 9.36 ± 4.02 years, respectively. TD children were reported to use both adaptive and maladaptive coping strategies more frequently than their peers with SEND (*p* ≤ 0.001). Parents also reported TD children as having higher coping efficacy (*p* < 0.001) for all reported coping strategies. Multiple factors were associated with higher odds of adaptive coping, including higher parental educational level, children's anxiety levels at the start of the pandemic, and their awareness of COVID-19. These factors were similarly associated with higher maladaptive coping and coping efficacy.

**Conclusion:**

TD children utilized a larger repertoire of coping strategies and had greater coping efficacy compared to peers with SEND early during the pandemic. These findings emphasize the need for targeted, community-level interventions to promote coping in children with SEND, particularly during pandemics and other public health crises.

## 1 Introduction

Since its declaration as a pandemic in March 2020, COVID-19 has profoundly affected individuals worldwide, especially children who faced significant disruptions in their daily lives (1–3). These disruptions included precautionary measures such as masking, social distancing, mandatory lockdowns, and prolonged periods of distance learning (4–6). In Saudi Arabia, children returned to in-person schooling after two years from the start of the pandemic, a lengthy period that presented significant challenges for caregivers and policymakers aiming to balance health precautions with educational and developmental needs (7, 8).

Globally, studies have consistently linked the pandemic to negative mental health outcomes across various populations (9–12), including children of different age groups (13, 14). The psychological burden of COVID-19 has prompted investigations into the predictors of emotional distress under such exceptional circumstances (15–17). Emotional distress refers to a psychological state marked by negative emotional responses, such as anxiety, fear, or sadness, triggered by stressful life events, including global health crises. Coping, which encompasses the cognitive and behavioral efforts used to manage such stressors as a worldwide pandemic, has been found to play a critical role in shaping mental health outcomes (18).

For children and adolescents, effective and adaptive coping strategies during the pandemic are thought to have been crucial, as these have been linked to good mental health outcomes (19–23). Maladaptive coping, in contrast, has been associated with poorer mental health during the pandemic. These findings and considerations have motivated efforts to study the coping strategies of children and adolescents during the pandemic (24–27).

Children with special educational needs and disabilities (SEND) have been a particularly vulnerable group during the pandemic (28–30). In addition to the common stressors faced by all children, those with SEND and their caregivers encountered disruptions to essential health and educational services (31–35). The prolonged duration of these challenges has highlighted gaps in support systems for this population.

Despite increased research efforts, several questions remain about the predictors of coping in children with SEND. We have previously reported on the pandemic's impact on children with SEND and their caregivers in Saudi Arabia, finding that their anxiety levels increased during the COVID-19 pandemic (36). Complementing that work, our aim in this study was to explore the coping strategies of children with SEND and compare them to typically developing (TD) children using their parents' reports. In addition, we aimed to identify the factors associated with more effective coping during the COVID-19 pandemic in this population.

## 2 Methods

### 2.1 Type of study

This was a cross-sectional survey and part of a global effort to investigate the mental health of individuals with disabilities during the COVID-19 pandemic. The study involved 60 researchers from over 30 countries (www.specialneedscovid.org) (35). Data was collected between May and July 2020 through an online survey distributed to caregivers (mainly parents) of children with SEND. Other details of study design have been reported elsewhere (36).

### 2.2 Participants

The study targeted caregivers of children with SEND in Saudi Arabia. Participants were recruited from among all registered beneficiaries of the Ministry of Human Resources and Social Development, the Autism Center of Excellence, and the Authority for Persons with Disabilities. Inclusion criteria were: (1) being a caregiver of a child aged 1–18 years; (2) having a child formally diagnosed with a special educational need or disability; and (3) living in a household that also includes a TD child. Caregivers who were unable to complete the questionnaire in Arabic were excluded. In total, caregivers provided data for 1,096 children.

### 2.3 Instruments

This study utilized the Arabic translation of the survey developed for the global project exploring the mental health of children with disabilities during the COVID-19 pandemic (35). The survey is accessible online via the OSF website (https://osf.io/5nkq9/). It has been previously employed to investigate the global burden of anxiety, concerns, and emotion regulation among individuals with Williams syndrome and Down syndrome during the COVID-19 pandemic (37). The psychometric properties and components of the Arabic survey have been detailed in our other report (36). The dataset for this study included parent-reported sociodemographic characteristics of households of children with SEND and TD children, as well as data on anxiety levels at various time points relative to the pandemic, coping strategy scores, and coping efficacy during the pandemic as reported by the children's parents.

### 2.4 Procedure

Participants were recruited through text message invitations sent to all beneficiaries of the Ministry of Human Resources and Social Affairs, the Autism Center of Excellence, and the Authority for Persons with Disabilities. The survey took around 35 min to complete. To ensure data security and allow for withdrawal of anonymized data, caregivers were required to enter a unique identification code comprising their name and birthdate initials at the end of the survey. This code was also used to prevent duplicate responses. Moreover, to maintain data quality, an attentiveness check was included in the survey and participants who failed this check were excluded from the study.

### 2.5 Ethical consideration

The Institutional Review Board at King Saud University examined and authorized the investigations involving human subjects (approval #20/0065/IRB). The patients/participants submitted their written informed consent to participate in this study. All methods in the study were performed in accordance with the Code of Ethics of the World Medical Association (Declaration of Helsinki).

### 2.6 Data analysis

The dataset for children with SEND and TD children was restructured to facilitate within-household comparisons and account for the pandemic timeline. Children within the same household were matched by age (±3 years), resulting in a dataset of 1,096 (548 pairs of children).

Data analysis was conducted using SPSS IBM Version 21, and the FACTOR standalone program was used for parallel analysis and scale validation. Descriptive statistics were used to summarize the data, with means and standard deviations describing continuous variables and frequencies and percentages describing categorical variables. We used the Kolmogrove-Smirnove test and Histograms to assess the normality of data distribution. Cronbach's alpha was used to assess internal consistency, while Exploratory Factor Analysis (EFA) and Parallel Analysis examined validity, factorial structure, and unidimensionality of psychometric measures.

For bivariate analysis, we used the non-parametric Mann-Whitney U test to compare measures between children with SEND and TD children. Spearman's correlation (rho) was used to assess the associations between continuous variables. The numeral scores were computed based on the results of our EFA of coping measures in children.

EFA with the Principal Components Analysis (PCA) and Parallel Analysis was applied to the to the correlation matrix of coping strategies of children as reported by their parents. We confirmed the sampling adequacy with a KMO index of 0.948 and Bartlett's test of sphericity indicated no multicollinearity (χ^2^ (91) = 45,287, *p* < 0.001) with a determinant value of 0.005. Two latent factors were identified, explaining 63.1% of the shared covariance between these predictors of coping strategies. The first factor, which explained 55.94% of the variance, was labeled “*adaptive coping*”. The second factor, which explained 8.01% of the variance, was labeled “*maladaptive coping”*. We subsequently performed Promax rotation due to a significant positive correlation between the factors (*r* = 0.683, *p* < 0.001). Appendix 1 details the factor solution for our measure of coping strategies.

Coping scores were dichotomized into “low” and “high” based on the sample mean due to skewness. We used Multivariate binary regression with Generalized Linear Mixed Models (GLMM) to evaluate the impact of relevant predictors on dichotomized coping strategies during the pandemic, with results presented as odds ratios (OR) and 95% confidence intervals (CI). The significance threshold was set at α = 0.05.

In the multivariable regression analysis, we evaluated school attendance status (whether students attended any educational setting before or during the pandemic) as a potential predictor of the outcome variables. Competing models were reviewed iteratively, and this variable did not demonstrate statistically significant relationships with the outcomes analyzed (*p* > 0.05). As a result, it was excluded from the final model. The final model was selected for its parsimony and relevance, including only those variables with established theoretical and statistical significance.

## 3 Results

### 3.1 Clinico-socio-demographic variables

The study included 548 families, each comprising a parent, a child with SEND, and a TD child from the same household for a total of 1,096 children. SEND children were matched with TD children of a similar age, with an age difference of no more than 3 years (±3 years). Therefore, the sample included 548 children with SEND and TD children of a similar age. Table 1 details the descriptive analysis of sociodemographic, health-related, and COVID-19-related variables for TD children and children with SEND. The descriptive analysis shows that most children in this sample, who were on average 9 years of age, were engaged in external activities such as attending school or daycare before the COVID-19 pandemic. However, during the pandemic, this shifted dramatically, with more than twice as many children remaining at home with their families. Among children with SEND, over 46% were reported by their parents as being unable to communicate their fears or anxieties. And whereas more than 80% of typically developing children were reported to be aware of COVID-19, less than one-third of children with SEND were aware of the pandemic's existence. Table 2 provides a descriptive analysis of the respondents' sociodemographic characteristics and household profiles.

**Table 1:** Sociodemographic, general health, and COVID-19-related variables for TD children and children with SEND, *N* = 1,096.

**Variable**	**Frequency (%)**	
	**TD children**	**Children with SEND**
**Sex**		
Male	306 (55.8%)	355 (64.8)
Female	242 (44.2)	193 (35.2)
**Age**		
(Mean Age in Years ± SD)	9.12 (3.95)	9.36 (4.02)
**Age group**		
1–2 years	23 (4.2)	16 (2.9)
3–6 years	131 (23.9)	124 (22.6)
7–10 years	194 (35.4)	206 (37.6)
>=11 years	200 (36.5)	202 (36.9)
**Primary diagnosis**		
Autism spectrum disorder	NA	142 (25.9)
Disorder of intellectual development	NA	89 (16.2)
Attention deficit hyperactivity disorder	NA	24 (4.4)
Developmental speech or language disorders	NA	17 (3.1)
Dyspraxia/developmental motor coordination disorder	NA	8 (1.5)
Other neurodevelopmental disorders	NA	200 (36.5)
Down syndrome	NA	66 (12)
Williams syndrome	NA	2 (0.4)
**History of intellectual disability**		
No	NA	170 (31)
Mild-moderate	NA	252 (46)
Severe	NA	126 (23)
**History of anxiety disorder**		
No	464 (84.7)	410 (74.8)
Yes	84 (15.3)	138 (25.2)
**Ability to communicate fear/anxiety**		
No, not at all	NA	254 (46.4)
Yes, nonverbally	NA	96 (17.5)
Yes, verbally & nonverbally	NA	198 (36.1)
**History of psychotropic medication use**		
No	NA	410 (74.8)
Yes	NA	138 (25.2)
**Diagnosed co-morbid health problems**		
No	489 (89.2)	174 (31.8)
Yes	59 (10.8)	374 (68.2)
**Allergies**		
No	NA	412 (75.2)
Yes	NA	136 (24.8)
**Living situation before COVID-19**		
At home with family	547 (99.8)	526 (96)
Supported living	0	1 (0.2)
With significant other	1 (0.2)	1 (0.2)
**Education/work before COVID-19**		
Preschool	23 (4.2)	10 (1.8)
Special education	37 (6.8)	94 (17.2)
Mainstream school	268 (48.9)	87 (15.9)
Daycare	9 (1.6)	116 (21.2)
Working	0	4 (0.7)
At home	207 (37.8)	227 (41.4)
Other	4 (0.7)	10 (1.8)
**Education/work during COVID-19**		
Preschool	4 (0.7)	2 (0.4)
Mainstream school	79 (14.4)	28 (5.1)
Daycare	9 (1.6)	12 (2.2)
At home	440 (80.3)	475 (86.7)
Other	4 (0.7)	9 (1.6)
**Child's awareness Of COVID-19**		
No	98 (17.9)	378 (69)
Yes	450 (82.1)	170 (31)
**History of suspected or confirmed infection with COVID-19**		
No	539 (98.7)	537 (98)
Yes	9 (1.6)	11 (2)

**Table 2:** Sociodemographic characteristic of survey respondents, *N*=548.

**Variable**	**Frequency**	**Percentage**
**Respondent's sex**		
Female	174	31.8
Male	374	68.2
**Respondent's (parent) age**		
(Mean age in years ± SD)		40.43 ± 7.43
**Age group**		
20–30 years	46	8.4
31–40 years	257	46.9
41–50 years	198	36.1
51–60 years	47	8.6
**Educational level**		
High school or less	480	43.8
Vocational degree/diploma	40	3.6
University degree	502	45.8
Higher studies	74	6.8
**Employment Status**		
Full-time paid job	336	61.3
Part-time paid job	28	5.1
Volunteering job	3	0.5
Prime homemaker	100	18.2
Unemployed	27	4.9
Student	4	0.7
Retired	34	6.2
Other	16	2.9
**Number of household members**		
1–3 persons	101	18.4
4–7 persons	287	52.4
>7 persons	65	11.9
Child lives elsewhere	95	17.3
**Residence prior to COVID-19**		
City/urban	426	77.7
Suburban/town	54	9.9
Rural/village	68	12.4
**Residence in Saudi Arabia**		
Central Region	432	40.2
Eastern Provinces	175	16.3
Western Provinces	282	26.3
Northern Provinces	77	7.2
Southern Provinces	108	10.1
**History of anxiety disorder (parent)**		
No	351	64.1
Yes	149	27.2
Prefer not to answer	48	8.8

### 3.2 Parents' concerns and worries around COVID-19

Respondents were surveyed about their level of anxiety or concern for their family's safety when they first heard about the COVID-19 pandemic. Responses were measured using a 7-point Likert scale, with a mean score of 3.25 (SD = 1.37), indicating moderate concern overall. The distribution of responses revealed that 29% of respondents reported feeling “moderately concerned,” while 25.9% reported being “very concerned.” A smaller proportion of respondents indicated either “high concern” (16.6%) or “mild concern” (13.7%), while 14.8% reported feeling “not concerned at all.” Moreover, respondents were surveyed on their level of anxiety or concern for their family's safety when they first heard about social distancing measures. Responses were similarly recorded on a 7-point Likert scale, with a mean score of 3.04 (SD = 1.31). The distribution of responses indicated that 15.5% were “not concerned at all,” 18.2% were “mildly concerned,” 31.4% were “moderately concerned,” 16.1% were “highly concerned,” and 18.8% were “very concerned.”

### 3.3 Children's anxiety levels around COVID-19

Respondents were asked to estimate their children's anxiety levels at various time points during the pandemic using a 5-point Likert scale. The detailed responses are presented in Table 3.

**Table 3:** Reported level of anxiety and worry in TD children and children with SEND around COVID-19, *N* = 1,096.

**Variable**	**Mean (SD)**		
	**Before COVID-19**	**At the start of the pandemic**	**Now**
**Children with SEND**, ***N*** = **548**			
1. How anxious is/was your child?	1.73 (1.26)	1.95 (1.37)	2.14 (1.44)
2. How concerned is/was your child about illness in general?	1.64 (1.22)	1.73 (1.26)	1.83 (1.34)
3. How concerned is/was your child about COVID-19?	1.60 (1.19)	1.74 (1.28)	1.79 (1.29)
4. How concerned is/was your child about your family's safety with respect to COVID-19?	1.57 (1.16)	1.70 (1.25)	1.81 (1.32)
5. How concerned is/was your child about their own health?	1.63 (1.20)	1.75 (1.28)	1.84 (1.34)
6 How concerned is/was your child about not being able to meet peers and friends?	1.87 (1.38)	2.01 (1.42)	2.15 (1.51)
7 How concerned is/was your child about not being able to approach others?	1.79 (1.33)	1.94 (1.40)	2.07 (1.50)
8 How concerned is/was your child about their loss of routine?	1.94 (1.37)	2.20 (1.43)	2.29 (1.51)
9 How concerned is/was your child about boredom?	2.01 (1.48)	2.20 (1.51)	2.35 (1.59)
10 How concerned is/was your child about the possibility of getting ill?	1.66 (1.26)	1.75 (1.30)	1.82 (1.36)
11 How concerned is/was your child about the possibility of others getting ill?	1.58 (1.17)	1.67 (1.21)	1.76 (1.27)
12 How concerned is/was your child about the loss of institutional support (e.g., school, workplace), including interventions (language therapist, psychologist, etc.)?	1.78 (1.36)	1.93 (1.40)	2.03 (1.51)
13. How concerned is/was your child about family conflict?	1.67 (1.27)	1.72 (1.30)	1.73 (1.32)
14. How concerned is/was your child about their financial/economic situation?	1.60 (1.24)	1.67 (1.29)	1.75 (1.38)
**TD children**, ***N*** = **548**			
1. How anxious is/was your child?	2.01 (1.30)	2.39 (1.37)	2.67 (1.43)
2. How concerned is/was your child about illness in general?	1.96 (1.30)	2.39 (1.35)	2.61 (1.43)
3. How concerned is/was your child about COVID-19?	1.96 (1.32)	2.39 (1.37)	2.66 (1.42)
4. How concerned is/was your child about your family's safety with respect to COVID-19?	2.10 (1.37)	2.48 (1.39)	2.66 (1.45)
5. How concerned is/was your child about their own health?	2.02 (1.35)	2.36 (1.38)	2.59 (1.46)
6 How concerned is/was your child about not being able to meet peers and friends?	2.24 (1.47)	2.64 (1.45)	2.79 (1.50)
7 How concerned is/was your child about not being able to approach others?	2.17 (1.43)	2.63 (1.43)	2.78 (1.47)
8 How concerned is/was your child about their loss of routine?	2.11 (1.41)	2.60 (1.43)	2.71 (1.50)
9 How concerned is/was your child about boredom?	2.24 (1.43)	2.70 (1.44)	2.84 (1.50)
10 How concerned is/was your child about the possibility of getting ill?	2.02 (1.4)	2.42 (1.45)	2.55 (1.45)
11 How concerned is/was your child about the possibility of others getting ill?	2.03 (1.35)	2.40 (1.38)	2.54 (1.43)
12 How concerned is/was your child about the loss of institutional support (e.g., school, workplace), including interventions (language therapist, psychologist, etc.)?	1.84 (1.29)	2.13 (1.40)	2.24 (1.46)
13. How concerned is/was your child about family conflict?	1.85 (1.31)	2.00 (1.35)	2.13 (1.43)
14. How concerned is/was your child about their financial/economic situation?	1.83 (1.32)	1.97 (1.38)	2.10 (1.47)

SEND, special educational needs and disabilities; TD, typically developing.

### 3.4 Children's copying strategies during the COVID-19 pandemic

Table 4 presents the bivariate analysis of children's use of various coping strategies during the pandemic, as reported by their parents on a 5-point Likert scale, where higher scores correspond to more frequent use of these coping strategies. TD children were reported to use most coping strategies more frequently than their peers with SEND. However, no significant differences were found in the use of aggression, isolation, or repetitive behaviors between the two groups (*p* > 0.05). Similarly, the parents' reported efficacy of each coping strategy during the COVID-19 pandemic, reported on a 5-point Likert scale, was significantly higher for TD children when compared to children with SEND, except for efficacy of coping by aggression (*p* = 0.086) as detailed in Table 5.

**Table 4:** Use of coping strategies by children with SEND compared to TD peers during the COVID-19 pandemic, *N* = 1,096.

**Variable**	**Reported use of coping strategy**		***z*-Value**	***p*-Value**
	**Mean** ^*^ **(SD)**			
	**Children with SEND**	**TD children**		
1. In order to feel less stressed, my child avoids any information about it	1.76 (1.29)	2.20 (1.40)	5.95	< 0.001
2. In order to feel less stressed, my child gets as much information as possible	1.86 (1.34)	2.34 (1.43)	8.8	< 0.001
3. In order to feel less stressed, my child talks about it as often as possible	1.79 (1.27)	2.22 (1.37)	6.16	< 0.001
4. In order to feel less stressed, my child distracts himself or herself as much as possible	1.81 (1.24)	2.29 (1.41)	7.04	< 0.001
5. In order to feel less stressed, my child changes the way he or she thinks about the situation	1.77 (1.22)	2.31 (1.40)	7.23	< 0.001
6. In order to feel less stressed, my child focuses on positive aspects or views the situation in a different light (e.g., having more family time together)	1.99 (1.39)	2.47 (1.48)	6.02	< 0.001
7. In order to feel less stressed, my child tells jokes and engages in humor	1.90 (1.34)	2.48 (1.50)	7.19	< 0.001
8. In order to feel less stressed, my child does not express negative emotions (i.e., suppression of emotions)	1.91 (1.30)	2.28 (1.37)	5.21	< 0.001
9. In order to feel less stressed, my child ruminates (i.e., thinks deeply about something)	1.87 (1.28)	2.28 (1.35)	5.91	< 0.001
10. In order to feel less stressed, my child engages in aggressive behaviors toward others around him or her	1.83 (1.630)	1.86 (1.25)	0.444	0.659
11. In order to feel less stressed, my child isolates himself or herself in his or her room or another room of the house	1.82 (1.32)	1.83 (1.22)	0.294	0.769
12. In order to feel less stressed, my child engages in repetitive behaviors (e.g., asking the same questions repetitively, repeatedly washing hands, rocking, or other stereotypic behaviors such as stimming)	2.01 (1.41)	2.16 (1.37)	1.83	0.067
13. I try to shield my child from the situation as much as possible	2.03 (1.47)	2.76 (1.60)	8.5	< 0.001
14. I try, or my child tries, to establish a routine in his or her daily life to lower the experienced stress	2.03 (1.40)	2.49 (1.47)	6.34	< 0.001

^*^Mean scores on a 5-point Likert scale where higher scores correspond to more frequent use of the specified coping strategy.

SEND, special educational needs and disabilities; TD, typically developing.

**Table 5:** Coping efficacy in children with SEND compared to TD peers during the COVID-19 pandemic, *N* = 1,096.

**Variable**	**Reported coping efficacy**		***z*-Value**	***p*-Value**
	**Mean** ^*^ **(SD)**			
	**Children with SEND**	**TD children**		
1. In order to feel less stressed, my child avoids any information about it	1.91 (1.39)	2.43 (1.47)	6.3	< 0.001
2. In order to feel less stressed, my child gets as much information as possible	2.00 (1.42)	2.55 (1.51)	6.25	< 0.001
3. In order to feel less stressed, my child talks about it as often as possible	1.90 (1.35)	2.44 (1.49)	7.03	< 0.001
4. In order to feel less stressed, my child distracts himself or herself as much as possible	1.90 (1.32)	2.43 (1.46)	7.3	< 0.001
5. In order to feel less stressed, my child changes the way he or she thinks about the situation	1.85 (1.29)	2.45 (1.46)	7.99	< 0.001
6. In order to feel less stressed, my child focuses on positive aspects or views the situation in a different light (e.g., having more family time together)	2.07 (1.44)	2.62 (1.52)	6.18	< 0.001
7. In order to feel less stressed, my child tells jokes and engages in humor	1.98 (1.38)	2.63 (1.54)	8.12	< 0.001
8. In order to feel less stressed, my child does not express negative emotions (i.e., suppression of emotions)	1.96 (1.33)	2.43 (1.41)	6.51	< 0.001
9. In order to feel less stressed, my child ruminates (i.e., thinks deeply about something)	1.97 (1.37)	2.45 (1.42)	6.5	< 0.001
10. In order to feel less stressed, my child engages in aggressive behaviors toward others around him or her	1.93 (1.41)	2.04 (1.38)	1.72	0.086
11. In order to feel less stressed, my child isolates himself or herself in his or her room or another room of the house	1.88 (1.35)	2.03 (1.36)	2.23	0.026
12. In order to feel less stressed, my child engages in repetitive behaviors (e.g., asking the same questions repetitively, repeatedly washing hands, rocking, or other stereotypic behaviors such as stimming)	2.08 (1.44)	2.35 (1.46)	3.31	0.001
13. I try to shield my child from the situation as much as possible	2.14 (1.53)	2.88 (1.61)	8.9	< 0.001
14. I try, or my child tries, to establish a routine in his or her daily life to lower the experienced stress	2.11 (1.45)	2.63 (1.51)	6.91	< 0.001

^*^Mean scores on a 5-point Likert scale where higher scores correspond to higher efficacy of the specified coping strategy.

SEND, special educational needs and disabilities; TD, typically developing.

When the children's average scores on the extracted factors of coping strategies were compared, TD children were found to have utilized both adaptive and maladaptive coping strategies significantly more frequently than children with SEND (*p* ≤ 0.001). Additionally, TD children were reported to have a significantly higher average coping efficacy during the pandemic (*p* < 0.001). The mean differences are presented in Table 6.

**Table 6:** Comparison of mean adaptive coping, maladaptive coping, and coping efficacy in children with SEND compared to TD peers during the COVID-19 pandemic, *N* = 1,096.

**Variable**	**Mean** ^*^ **(SD)**		**Mean difference (95% CI)**	***t*-Value/d.f**.	***p*-Value**

	**Children with SEND**	**TD children**			
Adaptive coping	1.89 (1.05)	2.32 (1.13)	−0.46 (−0.574: −0.360)	8.574/547	< 0.001
Maladaptive coping	1.97 (1.14)	2.15 (1.04)	−0.179 (−0.288: −0.0718)	3.27/547	0.001
Coping Efficacy	1.98 (1.07)	2.45 (1.13)	−0.472 (−0.575: −0.369)	9.04/547	< 0.001

^*^Mean scores on a 5-point Likert scale where higher scores correspond to more frequent use of coping strategies or higher efficacy of the specified coping strategy.

SEND, special educational needs and disabilities; TD, typically developing.

### 3.5 Predictors of children's coping strategies during the COVID-19 pandemic

Children's scores for adaptive coping, maladaptive coping, and coping efficacy were dichotomized into “high” and “low” categories based on the sample mean (0 = Low, 1 = High). We found that 47% of all children (*N* = 1096) had high adaptive coping scores, 50.7% had high maladaptive coping scores, and 51.1% had high coping efficacy scores. To explore the factors associated with higher odds of scoring “high” on each of these measures, we utilized multivariate logistic binary regression using Generalized Linear Mixed Models.

Table 7 presents the logistic regression model for predicting high adaptive coping in both TD children and children with SEND. The model revealed that higher parental education level, child's awareness of the pandemic, higher reported child anxiety at the start of the pandemic, higher pre-pandemic child anxiety, and higher current anxiety were each associated with an increased odds ratio (OR) for high adaptive coping. In contrast, a higher level of parental concern at the start of the pandemic was associated with a lower OR for adaptive coping in the child.

**Table 7:** Multivariate logistic binary regression analysis of odds for “High” adaptive coping among TD children and children with SEND during the COVID-19 pandemic, *N* = 1,096.

**Variable**	**Multivariate-adjusted OR**	**95% C.I. for OR**		***p*-Value**
		**Lower**	**Upper**	
Respondent's (parent) age	1.005	0.983	1.027	0.647
Respondent's (parent) educational level: university degree or higher education	1.197	1.070	1.340	0.002
(Child's) age	1.038	0.993	1.084	0.099
(Child's) sex: male	1.301	0.969	1.745	0.080
Diagnosed co-morbid health problems: Yes	1.452	0.987	2.134	0.058
Child's awareness of COVID-19: Yes	3.527	2.436	5.105	< 0.001
Group: TD children	1.489	0.992	2.236	0.055
How anxious is/was your child at the start of the pandemic	1.459	1.293	1.645	< 0.001
Respondent's (parent) concern level when he/she first heard of the pandemic	0.856	0.766	0.957	0.006
Respondent's (parent) anxiety level when he/she first heard of the pandemic	0.895	0.790	1.013	0.080
How anxious is/was your child before COVID-19	1.417	1.087	1.848	0.010
How anxious is/was your child now	1.342	1.071	1.682	0.011
Constant	0.005			< 0.001

Dependent variable = “Low” vs “High” Adaptive Coping Score.

SEND, special educational needs and disabilities; TD, typically developing.

Table 8 presents the regression model for predicting high maladaptive coping among all children. As with adaptive coping, child's awareness of COVID-19 was associated with a higher OR of maladaptive coping. Additionally, higher levels of reported child anxiety at the start of the pandemic and pre-pandemic (for children with SEND) were also associated with higher ORs of maladaptive coping. A history of anxiety disorder in the child and the child's ability to communicate fear or anxiety were both associated with higher ORs of maladaptive coping. As with adaptive coping, a higher level of parental concern at the onset of the pandemic was associated with lower ORs for maladaptive coping.

**Table 8:** Multivariate logistic binary regression analysis of odds for “High” maladaptive coping among TD children and children with SEND during the COVID-19 pandemic, *N* = 1,096.

**Variable**	**Multivariate-adjusted OR**	**95% C.I. for OR**		***p*-Value**
		**Lower**	**Upper**	
Respondent's (parent) age	0.998	0.977	1.018	0.826
Respondent's (parent) educational level: university degree or higher education	1.107	0.973	1.259	0.122
Respondent's (parent) employment status: Employed	0.759	0.566	1.017	0.065
(Child's) age	1.006	0.965	1.048	0.787
(Child's) sex: male	1.041	0.790	1.372	0.773
Diagnosed co-morbid health problems: Yes	1.255	0.881	1.788	0.207
Child's awareness of COVID-19: Yes	2.050	1.441	2.917	< 0.001
Group: TD children	1.402	0.956	2.055	0.084
How anxious is/was your child at the start of the pandemic	1.346	1.198	1.512	< 0.001
Respondent's (parent) concern level when he/she first heard of the pandemic	0.894	0.808	0.990	0.031
How anxious is/was your child before COVID-19 (Children with SEND)	1.553	1.318	1.829	< 0.001
Ability to communicate fear/anxiety: Yes, verbally & nonverbally	1.349	1.154	1.577	< 0.001
History of anxiety disorder: Yes	1.648	1.149	2.363	0.007
Constant	0.021			< 0.001

Dependent variable = “Low” vs “High” Maladaptive Coping Score.

SEND, special educational needs and disabilities; TD, typically developing.

Table 9 details the final model exploring factors associated with high coping efficacy for both TD and SEND children. Parents who were employed or had a higher level of education had a higher OR of reporting high coping efficacy in their children. Increasing child age and the child's awareness of COVID-19 were also associated with higher ORs of coping efficacy. Similarly, higher reported levels of child anxiety at the start of the pandemic and at the time of the survey were both linked to higher ORs of coping efficacy. As observed in the previous models, parental concern at the beginning of the pandemic was associated with a lower OR of coping efficacy. Notably, a history of intellectual disability was significantly associated with a reduced OR of coping efficacy.

**Table 9:** Multivariate logistic binary regression analysis of odds for “High” coping efficacy among TD children and children with SEND during the COVID-19 pandemic, *N* = 1,096.

**Variable**	**Multivariate-adjusted OR**	**95% C.I. for OR**		***p*-Value**
		**Lower**	**Upper**	
Respondent's (parent) age	0.990	0.967	1.013	0.385
Respondent's (parent) educational level: university degree or higher education	1.284	1.106	1.490	0.001
Respondent's (parent) employment status: Employed	0.633	0.454	0.883	0.007
(Child's) age	1.058	1.008	1.110	0.023
(Child's) sex: male	1.068	0.778	1.465	0.685
Diagnosed co-morbid health problems: Yes	1.120	0.738	1.700	0.593
Child's awareness of COVID-19: Yes	2.532	1.660	3.860	< 0.001
Group: TD children	1.024	0.634	1.656	0.922
How anxious is/was your child at the start of the pandemic	1.407	1.186	1.669	< 0.001
Respondent's (parent) concern level when he/she first heard of the pandemic	0.860	0.765	0.967	0.012
How anxious is/was your child before COVID-19	1.173	0.840	1.637	0.349
How anxious is/was your child now	1.135	0.967	1.332	0.121
How anxious is/was your child now (Children with SEND)	2.027	1.503	2.733	< 0.001
History of Intellectual Disability: Yes	0.563	0.397	0.799	0.001
History of anxiety disorder: Yes	1.210	0.800	1.831	0.366
Constant	0.036			< 0.001

Dependent variable = “Low” vs “High” Coping Efficacy Score.

SEND, special educational needs and disabilities; TD, typically developing.

## 4 Discussion

### 4.1 Overview of findings

This was a large nationwide study investigating the coping strategies of children with SEND as compared to their peers in the same household early during the COVID-19 pandemic. The present results complement our previous report on the rising levels of anxiety in children with SEND from before COVID-19 to the time of survey participation during the pandemic (36). Our current findings illustrate the impact of the pandemic on the rise in the proportion of children who stayed at home with their families during the pandemic as compared to before. We found that average anxiety levels increased in both children with SEND and TD children.

### 4.2 Comparison with the literature

Our comparative analysis demonstrated that TD children more frequently utilized various coping strategies and were reported to have higher coping efficacy compared to their peers with SEND. While direct comparative studies are scarce, this aligns with studies suggesting that children with SEN faced unique challenges during the COVID-19 pandemic, often exhibiting different coping strategies and mental health outcomes compared to their typically developing peers (38, 39).

This difference, however, was not observed for maladaptive coping strategies such as aggression, isolation, or repetitive behavior, as has been previously reported (26, 31). Given that children were of similar age in our analysis, the results demonstrate how TD children, when compared to peers with SEND, seemed to utilize coping strategies, whether judged to be adaptive or maladaptive, in a more efficacious manner. Strategies involving cognitive reappraisal, distraction, humor, and establishing routines were used more frequently by TD children and rated as more effective. This implies that interventions promoting these strategies may be particularly beneficial. Interestingly, aggressive coping was rated as similarly ineffective across both groups, reinforcing literature that positions externalizing behaviors as stress indicators rather than coping solutions (40, 41). The lower frequency and efficacy of coping strategies utilized by children with SEND may be attributed to their difficulties in applying skills such as appraising and actively responding to stressful situations, which are essential for developing healthy coping strategies (42).

### 4.3 Interpretation of the results

Multiple factors were found to be associated with higher odds of using adaptive coping strategies during the pandemic in this sample of children. These included household variables, such as the level of parental education, as well as individual child variables, such as being reported to have been more anxious at the start of the pandemic or having a comorbidity. Interestingly, these same factors were also found to predict higher odds of high scores on maladaptive coping and coping efficacy in the sample studied. This is likely because the coping strategy measures were interrelated, with the extracted factors being correlated despite loading on two separate components. Another explanation might be that certain factors, such as increasing child age, enable children to utilize a broader range of coping strategies.

A notable finding was that a child's awareness of the pandemic was significantly associated with higher odds of adaptive coping, maladaptive coping, and coping efficacy. This is consistent with prior research, as children who are aware of the pandemic, exhibit higher anxiety levels, or have a history of anxiety disorders appear to be at greater risk for maladaptive coping during the pandemic (43, 44). This might suggest a dual impact of children's awareness of the pandemic, and that our study participants didn't rely exclusively on one direction of coping styles. Instead, a mixture of behaviors has been utilized. Previous research has suggested that age-appropriate communication and education about the pandemic can improve and protect the psychological well being of children during the pandemic (45, 46).

High self-efficacy leads to greater effort, persistence, and resilience, even when facing obstacles (47), while low self-efficacy can result in avoidance or reduced effort (48), with applications in education influencing learning and achievement (49), health affecting treatment adherence (50), and coping shaping stress management, aligning with the finding of higher efficacy in typically developing (TD) children using maladaptive strategies, possibly due to strong self-belief. Theoretically, this suggests efficacy beliefs can mediate outcomes independently of strategy quality, as TD children might rely on maladaptive strategies (e.g., denial) yet achieve efficacy due to high self-efficacy bolstered by mastery experiences or social support (51), challenging linear coping models and proposing self-efficacy as a buffer for functional outcomes despite suboptimal methods, while also highlighting the need to assess and enhance self-efficacy in interventions, especially for populations with lower baseline confidence. In the context of our study, this theory could explain why TD children show higher coping efficacy, with their developmental advantages (e.g., better social modeling or emotional regulation) reinforcing self-efficacy, enabling maladaptive strategies to yield positive results (52), and further exploration could involve measuring self-efficacy alongside coping strategies to disentangle effects across developmental stages or support needs.

### 4.4 Limitations

This study has several limitations due to the utilized methodology and the structure of national SEND services. The reliance on an online cross-sectional survey may have excluded families with limited access to technology. Recruitment through disability service providers and support groups introduced potential selection bias, particularly underrepresenting rural and remote populations. Nevertheless, an important strength of the presented results was the national coverage achieved, with responses from all major regions of Saudi Arabia.

Another limitation is the reliance on parental reports as the primary data source, which may not have fully captured the experiences of older, verbal children with adequate cognitive skills. Reporting biases, such as the Horn effect, may have influenced perceptions of children's abilities, experiences, and behaviors during the pandemic. Future research should consider longitudinal designs and incorporate self-reported assessments by children where feasible.

Furthermore, the research relied on the same parent to provide data concerning both their child with a disability and their typically developing child. While this within-family comparison aided in controlling for variability in sociodemographic and parenting factors, it may have introduced shared-method variance or response bias. The parents' perspective might have influenced responses for both children in equivalent manners, potentially diminishing observed differences. Future studies may benefit from gathering data from diverse parent samples to enhance between-group comparisons. Moreover, a relatively high percentage of SEND and TD children were not attending any form of educational setting prior to the pandemic. This may have contributed to the observed lack of differences in maladaptive coping strategies between the two study groups. These children may not have experienced the same degree of social disruption or isolation as others, potentially influencing their coping responses. Given the observational nature of our research and its focus on a specific context, the findings may reflect similar educational and social dynamics in comparable settings. Future research should explore the role of pre-pandemic educational engagement in shaping coping strategies during public health crises.

The study's inclusion in an international project and use of secondary data limited flexibility, including a predefined list of variables. The broad age range of participants, from toddlers to adolescents, necessitates caution when interpreting our findings, given the diverse care needs across this spectrum. Lastly, the cross-sectional design restricts our ability to draw causal inferences or to capture changes in coping strategies over the course of the pandemic.

### 4.5 Implications of the findings

The findings from this study highlight the need for targeted, community-level interventions to enhance coping strategies, especially among children with SEND, particularly in times of crisis like the COVID-19 pandemic. The limited range of coping strategies observed in children with SEND indicates that interventions must emphasize personal skills and create a supportive, informed environment in families, schools, and communities.

School-based psychological support programs have shown promise in preventing challenging behavior and helping children develop adaptive coping skills in the face of anxiety and stress (53, 54). When implemented in schools or community centers, evidence-based programs such as the Coping Power Program and the Friends for Life program could provide children with SEND with structured opportunities to learn stress-management techniques, emotional regulation, and problem-solving skills in a safe, supportive environment (55, 56).

In addition, given the role of parental education in facilitating adaptive coping (as highlighted by our findings), community-based initiatives that aim to train parents and caregivers in strategies for supporting their children's emotional well being could be beneficial. Programs such as the *Incredible Years Parent Training Program* have effectively improved parents' ability to manage children's behavioral challenges and support emotional development. These programs could be adapted to include specific strategies for children with SEND, enhancing their resilience during stressful periods (55, 56). Also, parents of children (57).

Moreover, programs that promote social interaction, such as *Social Skills Training (SST)*, could help children with SEND build healthier coping strategies and more effective interpersonal interactions. By improving their social coping strategies, children may be better able to manage stress at home and in other settings (58).

Lastly, since our study found that children's awareness of the pandemic correlated with coping efficacy, community-wide initiatives that provide age-appropriate, accessible mental health education could enhance coping skills among children with SEND. For instance, community-wide campaigns could reduce stigma, increase awareness, and provide resources for children and families.

These interventions should be flexible and inclusive, considering the diverse needs of children with SEND. They should also involve multidisciplinary collaboration between educators, mental health professionals, and families. These interventions can play a critical role in enhancing coping skills and promoting overall well being by fostering an environment where children with SEND can access appropriate resources and support.

## 5 Conclusions

This nationwide study aimed to investigate the coping strategies of children with SEND compared to TD children during the COVID-19 pandemic in Saudi Arabia. Based on data from 548 families of children with SEND and their TD peers, we found that TD children more frequently employed a wider range of coping strategies and exhibited greater coping efficacy, as reported by their parents. Both children with SEND and TD children were reported to experience increased anxiety levels compared to before the pandemic.

However, TD children demonstrated more frequent use of both adaptive and maladaptive coping strategies, alongside higher overall coping efficacy during the pandemic. Factors such as parental educational level, children's anxiety levels at the onset of the pandemic, and their awareness of COVID-19 were associated with the use of a broader range of coping strategies and greater coping efficacy.

Our findings add to the growing international literature on the impact of COVID-19 on children with SEND. The results have important implications for policymakers seeking to support this vulnerable group in the post-pandemic period. Educators and parents should remain attentive to children's awareness of stressful events in their environment and provide them with accurate, age-appropriate information. Children with SEND require additional attention, especially those exhibiting increased anxiety during times of public health crises. To better meet the needs of children with SEND and their caregivers, we recommend the development of systematic and proactive community-level support initiatives that foster effective coping and address both their mental and general health needs, particularly during future emergencies.

## Data Availability

The raw data supporting the conclusions of this article will be made available by the authors, without undue reservation.

## Appendix

**Table A1:** Principal components analysis (Promax rotated) factor solution for children's coping strategies.

	**Extracted component**	
	**Adaptive coping**	**Maladaptive coping**
3. In order to feel less stressed, my child talks about it as often as possible	0.925	
2. In order to feel less stressed, my child gets as much information as possible	0.901	
1. In order to feel less stressed, my child avoids any information about it	0.843	
6. In order to feel less stressed, my child focuses on positive aspects or views the situation in a different light (e.g., having more family time together)	0.809	
5. In order to feel less stressed, my child changes the way he or she thinks about the situation	0.794	
7. In order to feel less stressed, my child tells jokes and engages in humor	0.772	
4. In order to feel less stressed, my child distracts himself or herself as much as possible	0.711	
9. In order to feel less stressed, my child ruminates (i.e., thinks deeply about something)	0.515	
8. In order to feel less stressed, my child does not express negative emotions (i.e., suppression of emotions)	0.470	
11. In order to feel less stressed, my child isolates himself or herself in his or her room or another room of the house		0.887
10. In order to feel less stressed, my child engages in aggressive behaviors toward others around him or her		0.859
12. In order to feel less stressed, my child engages in repetitive behaviors (e.g., asking the same questions repetitively, repeatedly washing hands, rocking, or other stereotypic behaviors such as stimming)		0.803
14. I try, or my child tries, to establish a routine in his or her daily life to lower the experienced stress		0.580
13. I try to shield my child from the situation as much as possible		0.532

## References

[1] Cucinotta D Vanelli M. WHO declares COVID-19 a pandemic. Acta Biomedica. (2020) 91:157–60. 10.23750/abm.v91i1.9397 32191675 PMC7569573

[2] Schnitzler L Janssen LMM Evers SMAA Jackson LJ Paulus ATG Roberts TE. The broader societal impacts of COVID-19 and the growing importance of capturing these in health economic analyses. Int J Technol Assess Health Care. (2021) 37:e43. 10.1017/S0266462321000155 33686927

[3] Ghosh R Dubey MJ Chatterjee S Dubey S. Impact of COVID-19 on children: Special focus on the psychosocial aspect. Minerva Pediatr. (2020) 72:226–35. 10.23736/S0026-4946.20.05887-9 32613821

[4] Araújo LA Veloso CF Souza M de C Azevedo JMC de Tarro G. The potential impact of the COVID-19 pandemic on child growth and development: a systematic review. J Pediatr. (2021) 97:369–77. 10.1016/j.jped.2020.08.008 32980318 PMC7510529

[5] Deoni SC Beauchemin J Volpe A D'Sa V Consortium the R. The COVID-19 pandemic and early child cognitive development: a comparison of development in children born during the pandemic and historical references. medRxiv. (2022) 2. 10.1101/2021.08.10.21261846 34401887 PMC8366807

[6] Chaabane S Doraiswamy S Chaabna K Mamtani R Cheema S. The impact of covid-19 school closure on child and adolescent health: a rapid systematic review. Children. (2021) 8:415. 10.3390/children8050415 34069468 PMC8159143

[7] Aljamaan F Alhaboob A Saddik B Bassrawi R Assiri R Saeed E. In-person schooling amidst children's COVID-19 vaccination: exploring parental perceptions just after omicron variant announcement. Vaccines. (2022) 10:768. 10.3390/vaccines10050768 35632524 PMC9147905

[8] Alhuzaimi AN Alrasheed AA Al-Eyadhy A Aljamaan F Alhasan K Batais MA. Exploring determinants of COVID-19 vaccine acceptance, uptake, and hesitancy in the pediatric population: a study of parents and caregivers in Saudi Arabia during the initial vaccination phase. Healthcare. (2023) 11:972. 10.3390/healthcare11070972 37046901 PMC10094388

[9] Leung CMC Ho MK Bharwani AA Cogo-Moreira H Wang Y Chow MSC. Mental disorders following COVID-19 and other epidemics: a systematic review and meta-analysis. Transl Psychiatry. (2022) 12:205. 10.1038/s41398-022-01946-6 35581186 PMC9110635

[10] de Sousa GM Tavares VDO de Meiroz Grilo MLP Coelho MLG de Lima-Araújo GL Schuch FB. Mental health in COVID-19 pandemic: a meta-review of prevalence meta-analyses. Front Psychol. (2021) 12:703838. 10.3389/fpsyg.2021.703838 34621212 PMC8490780

[11] Robinson E Sutin AR Daly M Jones A. A systematic review and meta-analysis of longitudinal cohort studies comparing mental health before versus during the COVID-19 pandemic in 2020. J Affect Disord. (2022) 296:567–76. 10.1016/j.jad.2021.09.098 34600966 PMC8578001

[12] Wu T Jia X Shi H Niu J Yin X Xie J. Prevalence of mental health problems during the COVID-19 pandemic: a systematic review and meta-analysis. J Affect Disord. (2021) 281:91–8. 10.1016/j.jad.2020.11.117 33310451 PMC7710473

[13] Almhizai RA Almogren SH Altwijery NA Alanazi BA Al Dera NM Alzahrani SS. Impact of COVID-19 on children's and adolescent's mental health in Saudi Arabia. Cureus. (2021) 13:e19786. 10.7759/cureus.19786 34963826 PMC8695694

[14] Elharake JA Akbar F Malik AA Gilliam W Omer SB. Mental health impact of COVID-19 among children and college students: a systematic review. Child Psychiatry Hum Dev. (2023) 54:913–25. 10.1007/s10578-021-01297-1 35013847 PMC8747859

[15] Meade J. Mental health effects of the COVID-19 pandemic on children and adolescents: a review of the current research. Pediatr Clin North Am. (2021) 68:945–59. 10.1016/j.pcl.2021.05.003 34538305 PMC8445752

[16] Asbury K Fox L Deniz E Code A Toseeb U. How is COVID-19 affecting the mental health of children with special educational needs and disabilities and their families? J Autism Dev Disord. (2021) 51:1772–80. 10.1007/s10803-020-04577-2 32737668 PMC7393330

[17] Vintila M Tudorel OI Stefanut A Ivanoff A Bucur V. Emotional distress and coping strategies in COVID-19 anxiety. Curr Psychol. (2023) 68:1–10. 10.1007/s12144-021-02690-8 35035193 PMC8744025

[18] Budimir S Probst T Pieh C. Coping strategies and mental health during COVID-19 lockdown. J Ment Health. (2021) 30:156–63. 10.1080/09638237.2021.1875412 33502917

[19] Girma A Ayalew E Mesafint G. Covid-19 pandemic-related stress and coping strategies among adults with chronic disease in Southwest Ethiopia. Neuropsychiatr Dis Treat. (2021) 17:1551–61. 10.2147/NDT.S308394 34045857 PMC8144169

[20] Khoury JE Atkinson L Bennett T Jack SM Gonzalez A. Coping strategies mediate the associations between COVID-19 experiences and mental health outcomes in pregnancy. Arch Womens Ment Health. (2021) 24:1007–17. 10.1007/s00737-021-01135-2 34145499 PMC8213535

[21] AlHadi AN Alarabi MA AlMansoor KM. Mental health and its association with coping strategies and intolerance of uncertainty during the COVID-19 pandemic among the general population in Saudi Arabia: cross-sectional study. BMC Psychiatry. (2021) 21:1–13. 10.1186/s12888-021-03370-4 34320930 PMC8317145

[22] Croghan IT Chesak SS Adusumalli J Fischer KM Beck EW Patel SR. Stress, resilience, and coping of healthcare workers during the COVID-19 pandemic. J Prim Care Community Health. (2021) 12:21501327211008448. 10.1177/21501327211008448 33834900 PMC8040550

[23] Labrague LJ. Psychological resilience, coping behaviours and social support among health care workers during the COVID-19 pandemic: a systematic review of quantitative studies. J Nurs Manag. (2021) 29:1893–905. 10.1111/jonm.13336 33843087 PMC8250179

[24] Domínguez-Álvarez B López-Romero L Isdahl-Troye A Gómez-Fraguela JA Romero E. Children coping, contextual risk and their interplay during the COVID-19 pandemic: a spanish case. Front Psychol. (2020) 11:577763. 10.3389/fpsyg.2020.577763 33391095 PMC7772313

[25] Orgilés M Morales A Delvecchio E Francisco R Mazzeschi C Pedro M. Coping behaviors and psychological disturbances in youth affected by the COVID-19 health crisis. Front Psychol. (2021) 12:565657. 10.3389/fpsyg.2021.565657 33828499 PMC8019796

[26] Zainel AA Qotba H Al-Maadeed A Al-Kohji S Al Mujalli H Ali A. Psychological and coping strategies related to home isolation and social distancing in children and adolescents during the covid-19 pandemic: cross-sectional study. JMIR Form Res. (2021) 5:e24760. 10.2196/24760 33851577 PMC8083952

[27] Vallejo-Slocker L Sanz J García-Vera MP Fresneda J Vallejo MA. Mental health, quality of life and coping strategies in vulnerable children during the COVID-19 pandemic. Psicothema. (2022) 34:249–58. 10.7334/psicothema2021.467 35485538

[28] Patel K. Mental health implications of COVID-19 on children with disabilities. Asian J Psychiatr. (2020) 54:102273. 10.1016/j.ajp.2020.102273 32653852 PMC7330593

[29] Tso WWY Chan KL Lee TMC Rao N Lee SL Jiang F. Mental health & maltreatment risk of children with special educational needs during COVID-19. Child Abuse Negl. (2022) 130:105457. 10.1016/j.chiabu.2021.105457 35033372 PMC8743505

[30] Aishworiya R Kang YQ. Including children with developmental disabilities in the equation during this COVID-19 pandemic. J Autism Dev Disord. (2021) 51:2155–58. 10.1007/s10803-020-04670-6 32816170 PMC7438977

[31] Theis N Campbell N De Leeuw J Owen M Schenke KC. The effects of COVID-19 restrictions on physical activity and mental health of children and young adults with physical and/or intellectual disabilities. Disabil Health J. (2021) 14:101064. 10.1016/j.dhjo.2021.101064 33549499 PMC7825978

[32] Narzisi A. Handle the autism spectrum condition during coronavirus (Covid-19) stay at home period: ten tips for helping parents and caregivers of young children. Brain Sci. (2020) 10:207. 10.3390/brainsci10040207 32244776 PMC7226467

[33] Holmes EA O'Connor RC Perry VH Tracey I Wessely S Arseneault L. Multidisciplinary research priorities for the COVID-19 pandemic: a call for action for mental health science. Lancet Psychiatry. (2020) 7:547–60. 10.1016/S2215-0366(20)30168-1 32304649 PMC7159850

[34] degli Espinosa F Metko A Raimondi M Impenna M Scognamiglio E. A model of support for families of children with autism living in the COVID-19 lockdown: lessons from Italy. Behav Anal Pract. (2020) 13:550–8. 10.31234/osf.io/48cme 32837696 PMC7266382

[35] Zhang S Hao Y Feng Y Lee NY. COVID-19 pandemic impacts on children with developmental disabilities: service disruption, transition to telehealth, and child wellbeing. Int J Environ Res Public Health. (2022) 19:3259. 10.3390/ijerph19063259 35328947 PMC8951004

[36] Alenezi S Temsah MH Alyahya AS Almadani AH Almarshedi A Algazlan MS. Mental health impact of COVID-19 on Saudi families and children with special educational needs and disabilities in Saudi Arabia: a national perspective. Front Public Health. (2022) 10:992658. 10.3389/fpubh.2022.992658 36238239 PMC9551570

[37] Sideropoulos V Sokhn N Palikara O Van Herwegen J Samson AC. Anxiety, concerns and emotion regulation in individuals with Williams syndrome and Down syndrome during the COVID-19 outbreak: a global study. Sci Rep. (2023) 13:8177. 10.1038/s41598-023-35176-7 37210403 PMC10199450

[38] Castle VE Sideropoulos V Jones CL Zhang D Van Herwegen J Palikara O. The impact of COVID-19 on the mental health and wellbeing of children with special education needs and disabilities: a systematic review. Rev J Autism Dev Disord. (2024). 10.1007/s40489-024-00453-2 PMC13582133 42756063

[39] Pozas M Letzel-Alt V. Coping with distance learning during COVID-19 and its impact on students' emotional experiences: differences between students with and without special education needs. J Res Spec Educ Needs. (2023) 23:354–64. 10.1111/1471-3802.12605

[40] Eisenberg N Spinrad TL Eggum ND. Emotion-related self-regulation and its relation to children's maladjustment. Annu Rev Clin Psychol. (2010) 6:495–525. 10.1146/annurev.clinpsy.121208.131208 20192797 PMC3018741

[41] Zimmer-Gembeck MJ Skinner EA. “The development of coping: implications for psychopathology and resilience”. In:Bornstein MH, editor. The Wiley Handbook of Developmental Psychology in Practice: Implementation and Impact. 1st ed. Hoboken, NJ: Wiley-Blackwell (2015). p. 1–61. 10.1002/9781119125556.devpsy410

[42] Shulman S Carlton-Ford S Levian R Hed S. Coping styles of learning disabled adolescents and their parents. J Youth Adolesc. (1995) 24:281–94. 10.1007/BF01537597

[43] Meherali S Punjani N Louie-Poon S Abdul Rahim K Das JK Salam RA. Mental health of children and adolescents amidst covid-19 and past pandemics: a rapid systematic review. Int J Environ Res Public Health. (2021) 18:3432. 10.20944/preprints202103.0149.v1 33810225 PMC8038056

[44] Sideropoulos V Dukes D Hanley M Palikara O Rhodes S Riby DM. The impact of COVID-19 on anxiety and worries for families of individuals with special education needs and disabilities in the UK. J Autism Dev Disord. (2022) 52:2656–69. 10.1007/s10803-021-05168-5 34196890 PMC8246131

[45] Dalton L Rapa E Stein A. Protecting the psychological health of children through effective communication about COVID-19. Lancet Child Adolesc Health. (2020) 4:346–47. 10.1016/S2352-4642(20)30097-3 32243784 PMC7270522

[46] Singh S Roy D Sinha K Parveen S Sharma G Joshi G. Impact of COVID-19 and lockdown on mental health of children and adolescents: a narrative review with recommendations. Psychiatry Res. (2020) 293:113429. 10.1016/j.psychres.2020.113429 32882598 PMC7444649

[47] Bandura A. Self-efficacy: toward a unifying theory of behavioral change. Psychol Rev. (1977) 84:191–215. 10.1037//0033-295X.84.2.191 847061

[48] Pajares F. Self-efficacy beliefs in academic settings. Rev Educ Res. (1996) 66:543–78. 10.3102/00346543066004543

[49] Schunk DH Pajares F. “The development of academic self-efficacy”. In:Wigfield A Eccles JS., editors. Development of Achievement Motivation. New York, NY: Academic Press (2002). p. 15–31. 10.1016/B978-012750053-9/50003-6

[50] Strecher VJ DeVellis BM Becker MH Rosenstock IM. The role of self-efficacy in achieving health behavior change. Health Educ Q. (1986) 13:73–92. 10.1177/109019818601300108 3957687

[51] Bandura A. Self-Efficacy: The Exercise of Control. San Diego, CA: W H Freeman and Company (1997).

[52] Schunk DH. Self-efficacy and achievement behaviors. Educ Psychol Rev. (1989) 1:173–208. 10.1007/BF01320134

[53] Frydenberg* E Lewis R Bugalski K Cotta A McCarthy C Luscombe-smith N. Prevention is better than cure: coping skills training for adolescents at school. Educ Psychol Pract. (2004) 20:117–34. 10.1080/02667360410001691053

[54] Durlak JA Weissberg RP Dymnicki AB Taylor RD Schellinger KB. The impact of enhancing students' social and emotional learning: a meta-analysis of school-based universal interventions. Child Dev. (2010) 82:405–32. 10.1111/j.1467-8624.2010.01564.x 21291449

[55] McDaniel SC Watkins L Chow JC Fedewa M Nemer S. Systematic literature review and meta-analysis of Coping Power: effects and implications for implementation. J Appl Sch Psychol. (2023) 39:350–74. 10.1080/15377903.2023.2196946

[56] Higgins E O'Sullivan S. “What works”: systematic review of the “FRIENDS for Life” programme as a universal school-based intervention programme for the prevention of child and youth anxiety. Educ Psychol Pract. (2015) 31:424–38. 10.1080/02667363.2015.1086977

[57] Gammon EA Rose SD. The Coping Skills Training Program for parents of children with developmental disabilities: an experimental evaluation. Res Soc Work Pract. (1991) 1:244–56. 10.1177/104973159100100302

[58] Spence SH. Social skills training with children and young people: Theory, evidence and practice. Child Adolesc Ment Health. (2003) 8:84–96. 10.1111/1475-3588.00051 32797550

